# Oxidized Low Density Lipoprotein and Inflammation in Gout Patients

**DOI:** 10.1007/s12013-013-9767-5

**Published:** 2013-09-26

**Authors:** Xingliang Jiang, Min Li, Qibin Yang, Lijun Du, Juan Du, Jingguo Zhou

**Affiliations:** 1 Department of Laboratory Medicine, the Affiliated Hospital of North Sichuan Medical College, Nanchong, 637000 China; 2 Institute of Rheumatology and Immunology, The Affiliated Hospital of North Sichuan Medical College, Nanchong, 637000 China

**Keywords:** Gout, Oxidized low density lipoprotein, Inflammatory cytokines

## Abstract

To analyze the levels of oxidized low density lipoprotein (ox-LDL) and inflammatory cytokines in the plasma of gout patients. The levels of ox-LDL, hypersensitive C-reactive protein (hs-CRP), interleukin-1β, interleukin-6 (IL-6) and tumor necrosis factor-α (TNF-α) were measured in the plasma of 41 gout patients [28 in acute phase episode, 13 in intermittent phase (IP)], and in 40 healthy controls. The relationship between ox-LDL and inflammation was also explored by measuring the levels of several pro-inflammatory cytokines in the plasma. The plasma levels of ox-LDL, hs-CRP, IL-6 and TNF-α were significantly increased in patients with gout in the acute phase compared to those in the IP group and healthy controls (*P* < 0.05), but the levels of TGF-β were significantly lower in the acute phase group than in the IP group and healthy controls (*P* < 0.01). The levels of ox-LDL in the gout patients in the IP were significantly higher than those in healthy controls (*P* < 0.05). Correlation analysis indicated that the levels of ox-LDL were positively correlated with hs-CRP, IL-6 and TNF-α (*r* = 0.343, *r* = 0.386, *r* = 0.659, *P* < 0.01, respectively), but negatively correlated with TGF-β levels in patients in the acute phase (*r* = −0.240, *P* < 0.05). The levels of ox-LDL in gout patients were significantly higher than those in healthy controls. The changes in ox-LDL levels may be associated with enhanced inflammation in gout patients.

## Introduction

Gout is a clinical syndrome caused by the deposition of sodium urate crystal in tissues or organs from supersaturated extracellular fluid. Asymptomatic hyperuricemia and acute arthritis are its most common clinical manifestations [1]. Epidemiological studies have found higher than expected levels of atherosclerotic disease in gout patients when compared with the general population. In particular, gout is associated with increased incidence of coronary heart disease and stroke. However, the exact pathological mechanism underlying this association is unclear [2].

Oxidized low density lipoprotein (ox-LDL) can promote the initiation and development of atherosclerosis (AS) through a variety of biological pathways. The serum levels of ox-LDL are an important predictor of the occurrence of atherosclerotic disease [3]. However, the levels of ox-LDL are affected by many factors.

The role of ox-LDL in AS formation has been well established. Tsutsumi, et al. [4] found that in patients with primary gout the concentration of plasma ox-LDL antibodies was significantly higher than in the normal population. Moreover, the concentration of plasma ox-LDL antibodies was also closely associated with the LDL particle diameter and HDL levels, indicating that the high incidence of atherosclerotic diseases in patients with gout might be closely related to increased ox-LDL levels. However, reports about the relationship between the level of ox-LDL and the inflammatory reaction in gout patients are rare.

When episodes of acute gouty arthritis happen, the crystallization of sodium uric acid triggers the activation of the lymphocytes of the joints and peri-articular lymphocytes, monocytes—macrophages as well other inflammatory cells. These cells in turn generate more pro-inflammatory cytokines such as interleukin-1β (IL-1β), interleukin-6 (IL-6) and tumor necrosis factor-α (TNF-α) in an autocrine or paracrine manner. These cytokines interact with each other and constitute a complex network leading to an enhanced inflammatory reaction [5].

In this study, we measured the concentrations of ox-LDL, IL-1β, IL-6, TNF-α, transforming growth factor-β (TGF-β) and the inflammatory marker hypersensitive C-reactive protein (hs-CRP) in gout patients to better understand the roles of those cytokines in the pathogenesis of gout and their mutual relationships, and to analyze the reasons underlying the high incidence of atherosclerotic diseases in gout patients.

## Materials and Methods

### Patients

Forty-one patients with gout were included from the outpatient and inpatient clinics in the Department of Rheumatology in Affiliated Hospital of North Sichuan Medical College from July 2009 to February 2010. All patients were diagnosed with gout according to the 1977 diagnostic criteria set forth by the American Rheumatism Association [6]. All patients were male with an average age of 45.3 ± 7.2 years (range: 22–66 year old). Based on the absence or presence of acute arthritis, patients with gout were divided into acute episode phase (AEP) groups and intermittent phase (IP) groups with 28 cases in the AEP group and 13 in the IP group. None of the patients had chronic renal failure, hypertension, diabetes, abnormal liver function, thyroid dysfunction or other complications. The control group was composed of 40 individuals who were all healthy male individuals from the medical examination center, Affiliated Hospital of North Sichuan Medical College. The average age was 46.7 ± 9.2 years (range: 21–68 years old) (not significantly different from the gout group, *P* = n.s.). CBC, urine analysis, liver and kidney function, abdomen and kidney ultrasounds were all normal in the control group. In addition, none of the individuals in the control group had cerebral vascular diseases, heart disease, diabetes, impaired glucose tolerance or liver disease.

### Methods

Five milliliter venous blood was taken from all patients in the morning without having breakfast. Heparin was used as anti-coagulant. Within 4 h, the plasma was separated and the biochemical parameters including uric acid, lipids, liver and kidney function, blood glucose and hs-CRP were measured. The hs-CRP levels were measured using the latex enhanced immune transmission turbidimetric method. All the plasma left was stored at −80 °C until the next measurement. The levels of ox-LDL, IL-1β, IL-6, TNF-α and TGF-β were measured by a double-antibody sandwich enzyme-linked immunosorbent assay (ELISA). The ELISA kits were purchased from Wuhan Youersheng Health Technology Inc. (Wuhan, Hubei, China).

### Statistics

Quantitative data are expressed as mean ± standard deviation (± SD). The differences between the groups were compared using the independent sample *t* test and analysis of variance. The correlation between two factors was calculated using linear correlation analysis. All the data were analyzed using SPSS13.0 statistical software and considered significant at a *P* < 0.05.

## Results

### Lipid Profiles in Gout Patients

We found that the levels of TC, LDL-C, ApoB and LP(a) were higher in patients in the two gout groups than in the control patients (Table 1, *P* < 0.05). We noted that the levels of HDL-C in patients in the AEP group were lower than those in the IP and control groups and this difference reached statistical significance (Table 1, *P* < 0.05). There was, however, no difference between the levels of HDL-C between the IP group and the control group (Table 1). The levels of TG are not significantly different between the three groups (Table 1).

**Table 1:** The levels of uric acid and lipid profile in each group (mean ± SD)

Group	Cases	UA (μmol/L)	TG (mmol/L)	TC (mmol/L)	HDL-C (mmol/L)	LDL-C (mmol/L)	ApoA (g/L)	ApoB (g/L)	LP(a) (mg/L)
AEP	28	524.7 ± 81.3^**#^	1.38 ± 0.79	4.87 ± 1.17^*^	0.84 ± 0.45^**#^	3.1 ± 1.78^**^	1.19 ± 0.56	0.85 ± 0.32^*^	253 ± 56^** #^
IP	13	472.5 ± 71.4^**^	1.45 ± 0.82	5.43 ± 1.45^**^	1.35 ± 0.53	2.97 ± 1.52^*^	1.31 ± 0.75	1.13 ± 0.46^*^	218 ± 89^*^
CTL	40	313.9 ± 51.3	1.32 ± 0.75	4.41 ± 0.87	1.37 ± 0.31	2.53 ± 1.03	1.21 ± 0.33	0.73 ± 0.14	167 ± 57

*AEP* acute gout attack group, *IP* intermittent episodes group, *CTL* control group

^*^ *P* < 0.05 and ^**^ *P* < 0.01 as compared with control group; ^#^ *P* < 0.05 as compared with IP group

#### ox-LDL, hs-CRP, IL-1β, IL-6, TNF-α and TGF-β Levels in Gout Patients

We found that the levels of plasma ox-LDL, hs-CRP, IL-6 and TNF-α in the AEP patient group were significantly higher than those in the IP group and the control group (Table 2, *P* < 0.05). The increases in hs-CRP are particularly significant with more than a threefold increase in hs-CRP in the AEP group relative to the IP and control group (Table 2).

**Table 2:** The levels of ox-LDL, hs-CRP, IL-1β, IL-6, TNF-α and TGF-β (mean ± SD)

Group	Cases	ox-LDL (μg/L)	hs-CRP (mg/L)	IL-1β (ng/L)	IL-6 (ng/L)	TNF-α (ng/L)	TGF-β (ng/L)
AEP	28	704.5 ± 185.7^**#^	31.2 ± 6.54^**#^	17.5 ± 7.9	53.6 ± 38.5^**#^	42.4 ± 27.7^*#^	18.8 ± 9.5^*#^
IP	13	677.9 ± 215.3^*^	8.54 ± 4.26	18.6 ± 9.7	38.8 ± 20.4	34.5 ± 17.6	35.8 ± 17.9
CTL	40	467.2 ± 160.1	7.07 ± 3.12	14.7 ± 8.3	35.4 ± 17.3	28.9 ± 13.5	32.7 ± 14.8

*AEP* acute gout attack group, *IP* intermittent episodes group, *CTL* control group

^*^ *P* < 0.05 and ^**^ *P* < 0.01 as compared with control group; ^#^ *P* < 0.05 as compared with IP group

We also found that the plasma levels of TGF-β in AEP patient group were almost half of those found in the IP patients and in the control groups (Table 2, *P* < 0.05). In the IP group the levels of ox-LDL were 1.45 fold higher compared to the control group (*P* < 0.05). The levels of hs-CRP in IP group were higher than that in patients from control group, there were no statistically significant differences (*P* *>* 0.05). The levels of IL-1β did not significantly differ between the groups (Table 2, *P* = n.s).

#### The Relationship Between ox-LDL and Cytokines and hs-CRP in Patients with Acute Gout

We measured the plasma levels of ox-LDL in patients with acute gout and found that there was a direct relationship between ox-LDL and the levels of hs-CRP (*r* = 0.343, *P* < 0.01), IL-6 (*r* = 0.386, *P* < 0.01), and TNF-α (*r* = 0.659, *P* < 0.01). In contrast, the levels of ox-LDL are negatively correlated with those of TGF-β (*r* = −0.240, *P* < 0.01).

## Discussion

We found that the concentrations of ox-LDL, IL-6, TNF-α and the inflammatory marker hs-CRP were significantly higher in acute gout attack patients than that in healthy controls or in the IP gout patients (*P* < 0.05), but the levels of TGF-β were significantly lower in the acute phase group than in the IP group and healthy controls (*P* < 0.05). Furthermore, the ox-LDL concentration is positively correlated with the levels of IL-6, TNF-α and hs-CRP, suggesting that the acute inflammatory reaction in acute gout patients might be associated with an increased level of ox-LDL. Studies have shown that gout flares occur as a result of these monosodium urate (MSU) crystal deposits in the joints being suddenly released and setting off an inflammatory cascade that manifests as an acute gouty arthritis attack [7, 8]. Pascual [9], found that MSU crystals remain in the SF (synovial fluid), causing persistent low-grade inflammation during the intercritical period. TNF-α and other pro-inflammatory cytokines in turn promote the release of macrophage oxygen-free radicals [10], inhibit antioxidant enzymes such as paraoxonase 1 (PON1) [11], and mediate the process by which HDL becomes inflammatory HDL [12]. The inflammatory HDL eventually is unable to reverse the transport of cholesterol and loses its role as an antioxidant eventually leading to an imbalance in cellular homeostasis [13]. LDL can easily be oxidized to ox-LDL in the oxidizing environment for a long time [14]. The increased concentration of plasma ox-LDL might be related to the increase of IL-6 and TNF-α leading to increased generation of peroxides in gout patients. In addition, the inflammatory marker C-reactive protein (CRP) might combine with lipoproteins and activate the complement system, leading to vessel intimal injury, massive release of oxygen-free radicals, enhanced lipid peroxidation and increased generation of ox-LDL [15]. Ox-LDL mediates vascular endothelial injury through various mechanisms and ultimately causes the occurrence and development of AS [16]. Animal experiments showed that TGF-β can inhibit the acute inflammation caused by MSU crystals through promoting macrophage differentiation, and effectively inhibit IL-1β and IL-6 synthesis and release [17]. In our study, we found that the levels of TGF-β were lower in the gout patients suggesting that there is an attenuation of the anti-inflammatory response that may result in an acceleration or prolongation of the inflammation in gout patients.

In our study, we also found that in patients with intermittent episodes of gout, the ox-LDL levels were significantly higher than that in control group. However, the inflammatory markers did not significantly increase in gout patients with intermittent episodes suggesting that in addition to inflammatory factors, there are other causes underlying the increase in the ox-LDL levels. The levels of serum uric acid in both acute and intermittent episodes gout patients were higher than that in the control patients. Several factors can lead to increases in uric acid that oxidize LDL. These include generation of free radicals through the reaction between uric acid and free radicals such as hydroxyl (OH^−^) and (ONOO^−^) [18, 19], the oxidation of LDL by uric acid in the presence of copper ions and lipid hydroperoxides [20], and indirectly through the activation of NADPH oxidase [21].

In inflammatory diseases, cellular damage results in the upregulation of xanthine oxidase (XO), leading to parallel increase of UA and free radicals production [22], leading to endothelial dysfunction [23]. Cytokines (TNF-α, IL-1, and IFN-γ) may increase the UA production via the upregulation of XO activity, and ROS-mediated cell damage [24, 25]. In this study, we did not find a correlation between uric acid levels and the levels of ox-LDL in gout patients, likely due to the normal fluctuations in uric acid. Single uric acid concentration measurements cannot accurately explain their influence on ox-LDL.

In the present study, we found that the main features of the lipid profile in gout patients is that the levels of TC, LDL-C, ApoB and Lp(a) were significantly increased and the plasma HDL-C levels were significantly decreased in acute episode gout patients, which are in line with the typical atherogenic lipid profile and similar to the results in Takahashi’s study [26]. This may be related to the fact that lipoproteins are involved in the regulation of cytokines that in turn play a powerful role in negatively regulating lipoproteins. In patients with gout, the pro-inflammatory cytokines such as IL-1β, IL-6 and TNF-α can reduce the synthesis and secretion of apolipoprotein in hepatocytes by affecting HMG-CoA reductase, leading to reduced levels of lipids and lipoprotein levels [27].

In summary, the concentrations of ox-LDL and pro-inflammatory cytokines IL-1β, IL-6 and TNF-α significantly increased in gout patients in our study, while TGF-β concentrations significantly decreased in gout patients relative to controls. Moreover, the concentration of ox-LDL is positively correlated with the levels of pro-inflammatory cytokines IL-1β, IL-6 and TNF-α suggesting an increased inflammatory response measured by the increased levels of pro-inflammatory cytokines IL-1β, IL-6 and TNF-α. This in combination with persistent hyperuricemia may underlie the increases in ox-LDL. The atherogenic sclerosing lipid profiles in gout patients are also significantly changed. In the acute inflammatory phase, the decline in blood lipids and lipoproteins might be related to the inflammatory cytokines. Increased ox-LDL levels, the inflammatory environment, the changes in lipid profile and the interaction among them might be the important reasons for the high incidence of AS disease in gout patients, prompting the active control of inflammation and uric acid levels in the body. Modifying the lipid profile may be useful in improving the long-term prognosis. The molecular mechanisms underlying the interactions between ox-LDL, uric acid and inflammation and their effect on the formation of AS still need to be further elucidated.
